# The Role of Polyphenolic Antioxidants from Tea and Rosemary in the Hydroxyl Radical Oxidation of *N*-Acetyl Alanine

**DOI:** 10.3390/molecules28227514

**Published:** 2023-11-10

**Authors:** Nikolaos Vagkidis, Jennifer Marsh, Victor Chechik

**Affiliations:** 1 Department of Chemistry, University of York, Heslington, York YO10 5DD, UK; nikolaos.vagkidis@org.chemie.uni-giessen.de; 2 Institute of Organic Chemistry, Justus Liebig University Giessen, Heinrich-Buff-Ring 17, 35392 Giessen, Germany; 3 The Procter & Gamble Company, Mason Business Center, 8700 Mason-Montgomery Road, Mason, OH 45040, USA; marsh.jm@pg.com

**Keywords:** polyphenolic antioxidants, tea extracts, rosemary extracts, peptide oxidation, mass spectrometry, isotope exchange

## Abstract

In dead biological tissues such as human hair, the ability of antioxidants to minimise autoxidation is determined by their chemical reactions with reactive oxygen species. In order to improve our understanding of factors determining such antioxidant properties, the mechanistic chemistry of four phenolic antioxidants found in tea and rosemary extracts (epicatechin, epigallocatechin gallate, rosmarinic and carnosic acids) has been investigated. The degradation of *N*-acetyl alanine by photochemically generated hydroxyl radicals was used as a model system. A relatively high concentration of the antioxidants (0.1 equivalent with respect to the substrate) tested the ability of the antioxidants to intercept both initiating hydroxyl radicals (preventive action) and propagating peroxyl radicals (chain-breaking action). LC-MS data showed the formation of hydroxylated derivatives, quinones and hydroperoxides of the antioxidants. The structure of the assignment was aided by deuterium exchange experiments. Tea polyphenolics (epicatechin and epigallocatechin gallate) outperformed the rosemary compounds in preventing substrate degradation and were particularly effective in capturing the initiating radicals. Carnosic acid was suggested to act mostly as a chain-breaking antioxidant. All of the antioxidants except for rosmarinic acid generated hydroperoxides which was tentatively ascribed to the insufficient lability of the benzylic C-H bond of rosmarinic acid.

## 1. Introduction

Products of plant origin have a long history of use in traditional and folk medicine. They exhibit a variety of properties including radical scavenging [1]. In recent years, this has led to a surge of interest in plant-derived antioxidants as many consumers show a preference for products that contain compounds derived directly from nature. It is therefore important to understand the mechanism of action of these materials. In living systems, biological activity of antioxidants is often determined by mechanisms not related to radical scavenging. Moreover, the potential of antioxidants to negatively influence health is often overlooked, and today there are multiple reports demonstrating that unnecessary antioxidant supplementation has been linked to damaging biomolecules [2,3,4,5,6]. On the other hand, in dead biological tissue, such as food, fabric and hair, the role of antioxidants is limited to chemical reactions and is easier to establish. Plant extracts have therefore found particularly successful applications in areas such as food, textiles and the cosmetic industry. For instance, natural antioxidants are used as food preservatives, and have been shown to increase the shelf-life of high-fat-content food [7,8,9]. In another example, the treatment of fabric (cotton and wool) with natural antioxidants significantly enhanced both the antibacterial and antioxidant properties of the fabric [10]. Finally, botanical extracts have been reported to offer colour, protein and growth protection to human hair [11,12,13,14]. In this paper, we explore the ability of antioxidants to inhibit chemical oxidation processes which may not be of relevance to living biological systems but are important for reducing oxidative damage in dead tissue such as hair or wool. The mechanisms of antioxidant action in such chemical systems are often underexplored, and reports in the literature usually focus on either antioxidant potency, or the products of antioxidant degradation [15,16,17,18]. Mechanistic studies of antioxidant action in the oxidation of model substrates are rare.

**Figure 1:**
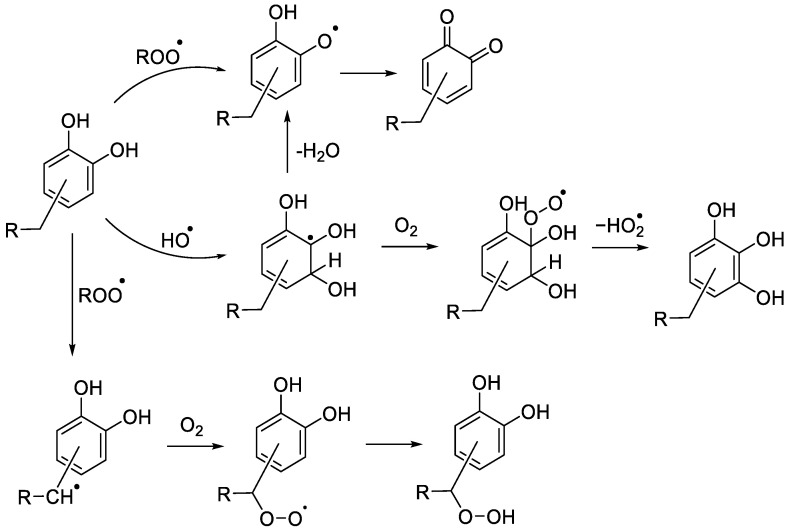
Reactions of polyphenols with hydroxyl and peroxyl radicals.

The antioxidant properties of botanical extracts can be attributed to the presence of several classes of compounds including polyphenols such as flavonoids and non-flavonoids (e.g., phenolic acids). Polyphenols act as antioxidants mostly due to their ability to scavenge radicals via hydrogen atom donation, as the corresponding phenoxyl radicals are stabilised by the aromatic ring and the adjacent hydroxyl substituents. For instance, the bond dissociation enthalpy (BDE) of hydroquinone is 81.2 kcal mol^−1^ vs. 87.6 kcal mol^−1^ for phenol [19,20,21,22]. Polyphenols therefore rapidly react with the peroxyl radicals ROO^•^ which are involved in the propagation step of the autoxidation reaction, thus breaking the radical chain reaction (Figure 1). However, polyphenols can also undergo other reactions with free radicals such as electron transfer or addition to the aromatic ring. For instance, the reaction of phenols with hydroxyl radical leads to further hydroxylation and the formation of polyphenols that have even lower BDEs of O-H bonds, and can, therefore, act as antioxidants in their own right (e.g., the BDE of the O-H bond in pyrogallol (1,2,3-trihydroxybenzene) is 75.3 kcal mol^−1^) [23].

**Figure 2:**
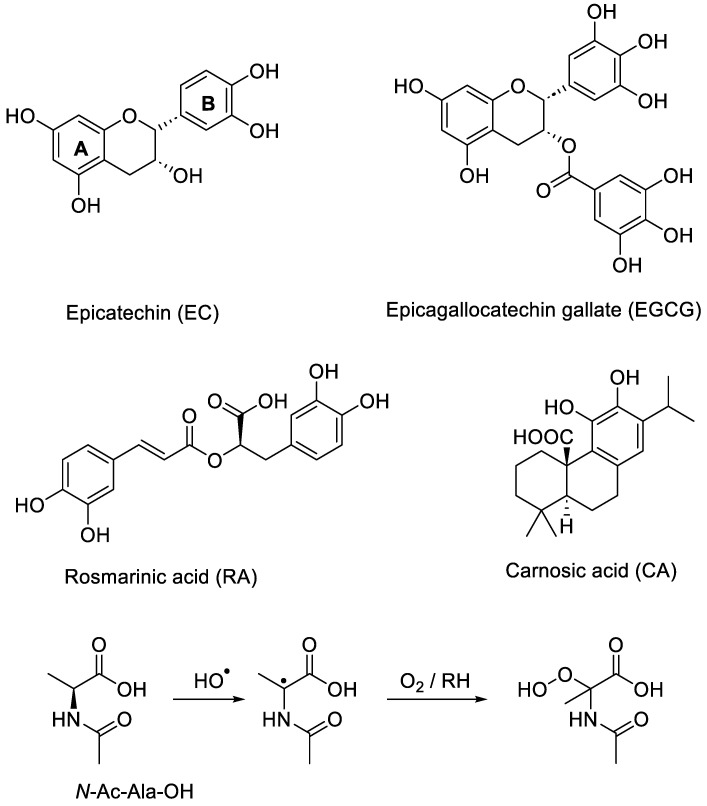
Chemical structures and abbreviations for the polyphenolic antioxidants and the proposed pathway for the substrate oxidation.

In order to interpret the antioxidant properties of botanical extracts, the reaction pathways of individual polyphenols need to be determined. Here, we report a mechanistic study of the antioxidant activity of four isolated antioxidants found in tea and rosemary (Figure 2). Most previous studies of these compounds investigated their ability to inhibit lipid peroxidation and quench ROO^•^ generated by azo initiators (e.g., AAPH or AIBN) [24,25,26,27]. Hydrogen atom abstraction from these compounds by ROO^•^ gives delocalised 1,2-semiquinone radicals, which can be further oxidised to the corresponding quinones. Quinones are indeed often identified as the main degradation product of the ROO^•^-mediated oxidation of flavonoids [28,29,30,31,32]. Autoxidation of the polyphenolics in the absence of substrates has also been shown to result in the corresponding quinones [30,33,34], and quinone formation was also reported in presence of transition metal ions (e.g., Cu^2+^) [5]. Apart from quinones, for EC and EGCG, there is some evidence to support the formation of dimers as a result of radical–radical reactions between two highly stabilised semiquinone radicals [35].

While autoxidation involves peroxyl radicals in the propagation stage, the initiating radicals are often hydroxyls. Hydroxyl radicals quickly (often close to the diffusion-controlled limit) react with most organic compounds with limited selectivity. It would, however, be a mistake to assume that hydroxyl radicals react with different functional groups with the same rate constants. For instance, hydroxyl radicals react with antioxidants ca. 100 times faster than with the least reactive peptides [36]. In most systems, antioxidants are present at much lower concentrations than other organic compounds, and antioxidants are, therefore, unlikely to intercept a significant proportion of hydroxyl radicals. However, in heterogeneous systems, the surface concentration of antioxidants can be relatively high, and therefore, their reactions with hydroxyl radicals may be non-negligible.

Reactions of polyphenolics with hydroxyl radical are underexplored. Although flavonoids are known to be powerful HO^•^ scavengers [37], there are only very few reports to support the formation of hydroxylated products from the oxidation of EC, EGCG, RA or CA [38,39]. In this work, we investigate the mechanism of action for the four antioxidants in aerobic HO^•^-induced oxidation of *N*-acetylated alanine. This compound can be used as a model for the degradation of proteins in dead biological tissue such as food, fabric or hair. *N*-Ac-Ala-OH will predominantly react with oxygen-centred radicals by donating a hydrogen atom from a C-H bond giving a stabilised tertiary radical which, after reaction with molecular oxygen, would yield a peroxyl radical and finally a hydroperoxide, a typical autoxidation mechanism (Figure 2). *N*-Ac-Ala-OH would be expected to react with hydroxyl radicals significantly slower than antioxidants [36]. We have previously reported the detection of hydroperoxides and alcohols (hydroperoxide degradation products) in hydroxyl-initiated *N*-Ac-Ala-OH degradation using mass spectrometry [40]. Here, the intermediates and products of antioxidant reactions are identified with electrospray ionisation mass spectrometry (ESI-MS).

## 2. Results and Discussion

### 2.1. Hydroxyl Radical-Induced Oxidation of N-Ac-Ala-OH

The oxidation of *N*-Ac-Ala-OH was initiated by the photolysis of hydrogen peroxide with a high-pressure Hg lamp which provides a broad-band UV output, in the presence of an antioxidant. The irradiation time was 2 min. All four antioxidants possess aromatic rings and hence absorb UV light. The UV-Vis spectra of pure antioxidants and *N*-Ac-Ala-OH, as well as the reaction mixtures before and after exposure to UV light can be found in the Supplementary Materials, Figures S1 and S2. To reduce the probability of direct photolysis of *N*-Ac-Ala-OH and antioxidants, hydrogen peroxide was used in large excess: the molar concentrations of *N*-Ac-Ala-OH, H_2_O_2_ and antioxidant were 1, 100 and 0.1 mM, respectively. Only ca. 0.3% of hydrogen peroxide decomposed under these conditions. The H_2_O_2_ decomposition was determined spectroscopically, by irradiating aqueous solutions of H_2_O_2_ (100 mM) and measuring its absorbance at 240 nm using ε_240 nm_ = 39.4 M^−1^, before and after exposure to the UV lamp [40]. A high concentration of antioxidants was necessary for the detection of their oxidation products as these compounds ionise very poorly in the positive mode ESI MS. Therefore, these compounds are commonly analysed in the negative mode ESI MS in the literature. However, the deuterium exchange experiments used in this work (vide infra) cannot be used in the negative mode due to partial deuterium incorporation into the aromatic rings of the deprotonated polyphenols [41]. Fortunately, LC-MS analysis in the positive ESI MS mode showed better sensitivity than direct injection of MS, probably due to the separation of the components in the reaction mixture that otherwise could have suppressed the ionization of polyphenols. Therefore, positive mode ESI MS was used for the product analysis. LC-MS chromatograms of the antioxidants are included in the Supplementary Materials, Figures S3–S6. In a control experiment, aqueous solutions (0.1 mM) of the antioxidants were exposed to UV light in the absence of the protected amino acids and H_2_O_2_, and their degradation was assessed spectroscopically by UV-Vis. No decomposition of the antioxidants, and no degradation products were detected after a 10 min irradiation, thus confirming the negligible role of direct photolysis. Under these conditions, and in the absence of antioxidants, we observed a ca. 11% degradation of *N*-Ac-Ala-OH.

### 2.2. Hydroxylation of Antioxidants and Quinone Formation

**Figure 3:**
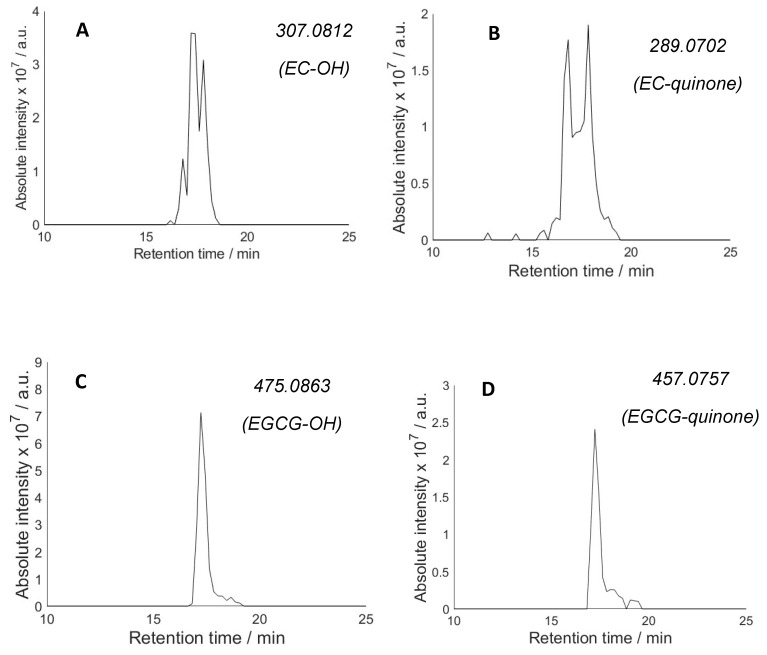

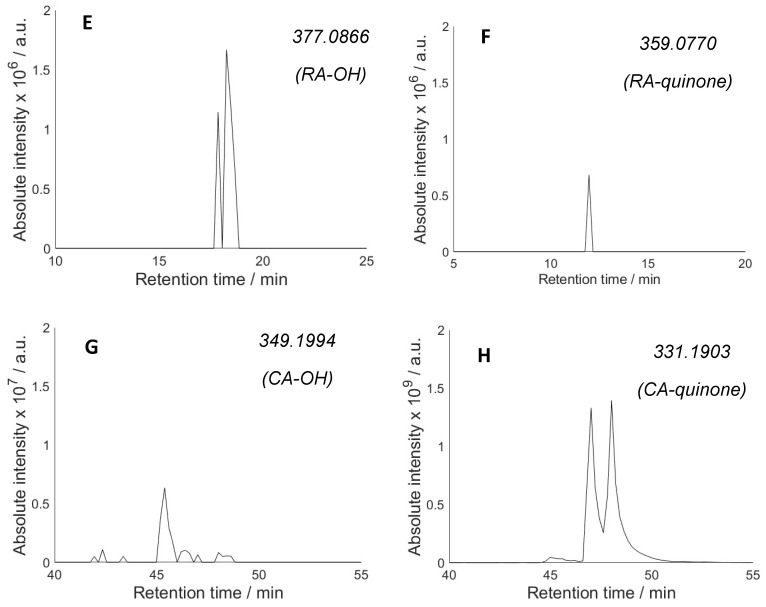
Extracted ion chromatograms of the [M + H]^+^ for (**A**) EC-hydroxylation, (**B**) EC-quinone), (**C**) EGCG-hydroxylation, (**D**) EGCG-quinone, (**E**) RA-hydroxylation, (**F**) RA-quinone, (**G**) CA-hydroxylation and (**H**) CA-quinone.

When the reaction mixtures containing H_2_O_2_, *N*-Ac-Ala-OH and antioxidants were analysed with LC-MS after photolysis, new peaks consistent with the formation of both hydroxylated polyphenols and quinone derivatives appeared in all cases (Figure 3). The products of further oxidation of these compounds have also been detected. The hydroxylation is most likely the result of the direct reaction of hydroxyl radical with antioxidants (via ^•^OH addition to the aromatic rings). Although the concentration of the antioxidant is 10 times lower than that of *N*-Ac-Ala-OH, the rate of hydroxyl radical addition to the aromatic ring is near the diffusion control limit (*k*~10^10^ M^−1^ s^−1^) [42], whereas hydrogen abstraction by hydroxyl radical from *N*-Ac-Ala-OH is ca. 2 orders of magnitude slower (*k*~10^8^ M^−1^ s^−1^) [43]. One would, therefore, expect the antioxidants to intercept a significant proportion of hydroxyl radicals. For some antioxidants (EC, RA), multiple peaks corresponding to the potential hydroxylated products were observed (Figure 3A,E). As these compounds were used as single isomers, the observation of several peaks suggests that hydroxylation occurred competitively at several positions in the antioxidant molecules yielding products with the same *m*/*z* ratio (vide infra).

For three antioxidants (EC, EGCG and RA), the hydroxylated product was detected in higher relative abundance than the quinone, whereas CA gives a significantly stronger quinone peak. While the ESI-MS ionisation efficiencies of different (even related) compounds could be substantially different and so peak intensities may not accurately reflect the relative concentrations, the higher hydroxylated product/quinone ratio for EC, EGCG and RA relative to CA may reflect the statistically larger number of available positions for hydroxylation in the former molecules. Hydroxylation of EC, EGCG and RA is expected to occur preferentially in the aromatic ring positions that are activated by the adjacent OH groups, consistent with the selective hydroxylation patterns reported for HO^•^-induced oxidation of dihydroxy benzenes [38]. Therefore, predominant formation of 4, 6, 4 and 1 different hydroxylated products can be predicted for EC, EGCG, RA and CA, respectively. The structures of these isomers are given in the Supplementary Materials, Figure S7. In agreement with the formation of multiple hydroxylated products for EC, EGCG and RA (Figure 3), multiple peaks were observed in extracted ion LC-MS chromatograms with the same *m*/*z* ratio. The number of peaks does not match the number of predicted isomers; this is likely due to incomplete separation of all isomeric derivatives under the LC-MS conditions used. CA shows only one hydroxylated product consistent with only one position available for hydroxylation.

**Figure 4:**
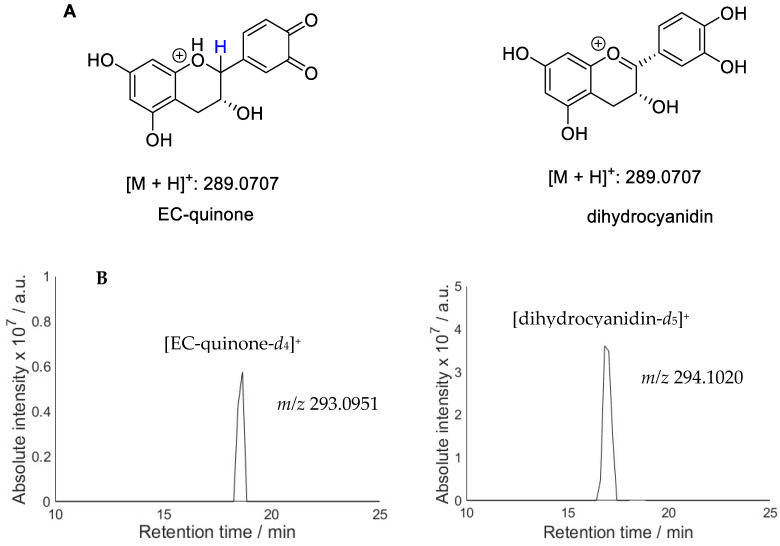
(**A**) Chemical structures and *m*/*z* for dihydrocyanidin and EC-quinone; (**B**) extracted ion chromatograms for deuterium exchange mixture of oxidised EC.

An analysis of the quinone products (Figure 3) also shows multiple products, e.g., two peaks for EC. Only one quinone is expected to form on the B ring of this molecule (Figure 2). However, a structure isomeric to quinone has previously been observed in the EC oxidation mixture and identified as dihydrocyanidin (Figure 4) [44]. In order to distinguish between the two potential structures, deuterium exchange was employed by conducting the photolysis experiment in D_2_O. Phenolic OH groups undergo rapid deuterium exchange whereas C-H protons do not participate in exchange under reaction conditions. Protonated EC-quinone has four labile protons while dihydrocyanidin possesses five labile protons. Complete exchange of OH with OD would therefore result in [M + D]^+^ *m*/*z* 294.1020 for dihydrocyanidin and [M + D]^+^ *m*/*z* 293.0951 for EC-quinone. Extracted ion chromatograms (Figure 4B) show that the two LC peaks correspond to compounds with a different number of exchangeable protons: the first LC peak belongs to dihydrocyanidin and the LC peak with the higher retention time can be assigned to EC-quinone.

**Figure 5:**
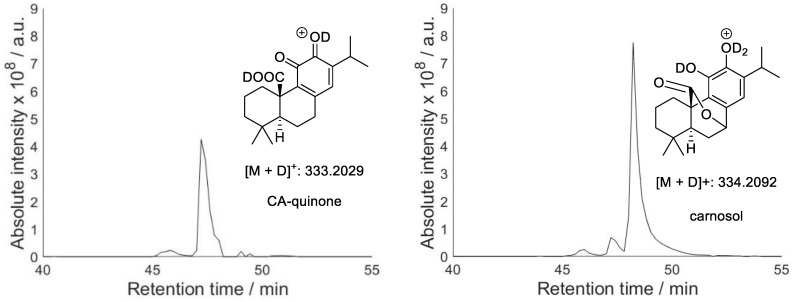
Structures and extracted ion chromatograms for deuterium exchange mixture of oxidised EC-quinone.

EGCG and RA can yield several different quinones with similar structures. The extracted ion chromatograms show a poorly resolved peak for EGCG and a single peak for RA. It is likely that the isomeric quinones cannot be separated under the LC conditions used. CA, on the other hand, gave two quinone peaks despite only one possible quinone product. Formation of CA quinone during CA oxidation has been reported [29]; however, CA is also known to oxidise to isomeric carnosol [30]. Carnosol and CA quinone possess a different number of exchangeable protons and hence their LC-MS peaks can be distinguished using deuterium exchange. Figure 5 shows extracted ion chromatograms for the deuterated CA quinone and carnosol. The results confirm formation of both products and made it possible to assign them to the corresponding LC-MS peaks.

Although MS peak intensities are affected by the ionisation efficiency, strong trends for similar compounds can be tentatively interpreted. For instance, the much higher relative intensity of quinone peak for CA as compared to the other antioxidants suggests that EC, EGCG and RA react mostly with hydroxyl radicals (yielding hydroxylated products) and act as preventive antioxidants under reaction conditions, whereas CA intercepts peroxyl radicals (yielding quinone) and hence acts as a chain-breaking antioxidant. Although quinones can form following an attack of either hydroxyl or peroxyl radical on the antioxidants (Figure 1), preferential formation of quinone is likely due to hydrogen abstraction by peroxyl radical. This is supported by control experiments carried out with hydrogen peroxide photolysis in the absence of *N*-Ac-Ala-OH substrate, which showed formation of hydroxylated derivatives but no quinones for all antioxidants.

### 2.3. Hydroperoxides of Antioxidants

**Figure 6:**
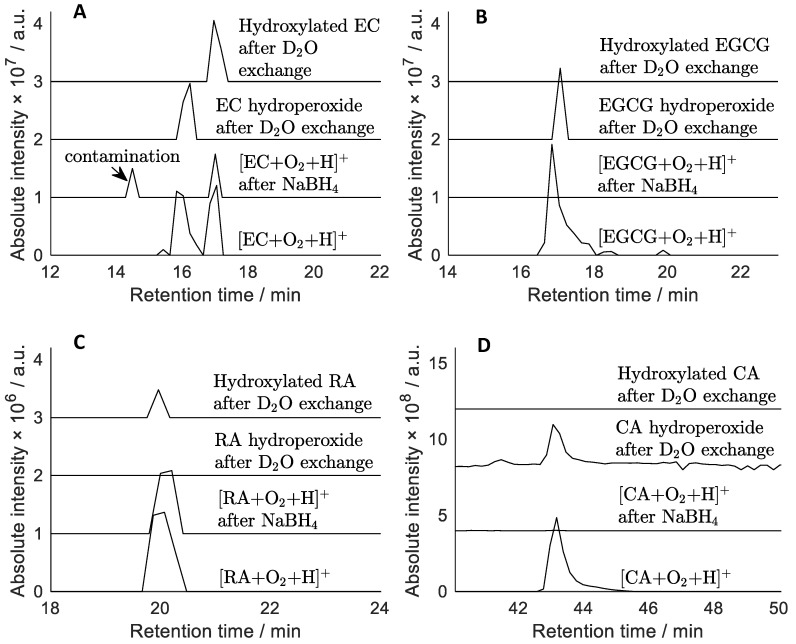
Extracted ion chromatograms for the [M + H]^+^ or [M + D]^+^ peaks of hydroperoxides or dehydroxylated derivatives of EC (**A**), EGCG (**B**), RA (**C**) and CA (**D**).

Apart from weak phenolic O-H bonds, the benzylic C-H bonds in catechins also have low BDE [44,45]. Abstraction of this C-H hydrogen, e.g., by a peroxyl or hydroxyl radical, yields a carbon-centred radical that will react with molecular oxygen, eventually forming a hydroperoxide. Understanding hydroperoxide formation of antioxidants is important not least because they can act as radical initiators in their own right. For instance, hydroperoxides can produce hydroxyl and alkoxyl radicals via thermal degradation or via electron transfer, e.g., with a redox-active transition metal ion [46]. Accumulation of hydroperoxides could, therefore, result in the pro-oxidant activity of antioxidants. Compounds with *m*/*z* ratios corresponding to the hydroperoxides of the antioxidants have been observed in LC-MS. However, unambiguous assignment of their structure is challenging as they are isomeric with compounds possessing two hydroxyl groups (e.g., HO-R-OH). In order to distinguish between such isomers, two approaches were used. In the first approach, oxidation mixtures were treated with sodium borohydride (NaBH_4_). This decomposes hydroperoxides via non-radical pathways, whereas hydroxylated derivatives are unaffected [47]. The disappearance of the LC-MS peak following NaBH_4_ treatment thus identifies it as a hydroperoxide. In the second method, deuterium exchange was used, as hydroxylated derivatives and isomeric hydroperoxides have different numbers of exchangeable protons. Following exchange, these compounds will have different mass; hence they can be identified from extracted ion chromatograms for the corresponding *m*/*z* values. The results of these experiments are shown in Figure 6.

### 2.4. Antioxidant Capacity

**Figure 7:**
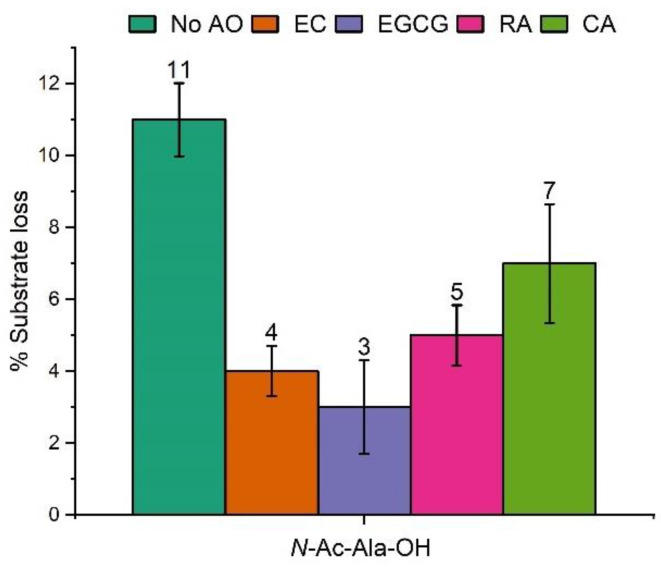
The % decomposition of *N*-Ac-Ala-OH upon exposure of aq. solutions (all 1 mM) to UV light for 2 min in presence of aq. H_2_O_2_ (100 mM), and the antioxidant (0.1 mM). Reactions were carried out in triplicate and the results are the mean ± SE of the experiments.

In order to assess the antioxidant properties of EC, EGCG, RA and CA under our reaction conditions (i.e., oxidation of *N*-Ac-Ala-OH by photolysis of H_2_O_2_), the decay of *N*-Ac-Ala-OH was monitored by LC-MS in the absence and presence of each compound (Figure 7). Photolysis conditions used in these experiments were designed so that, while the overall substrate loss was noticeable to enable meaningful analysis of antioxidant activity, the substrate degradation was not so high that the products of its oxidation would not have a major effect on the chemistry.

The results show that all antioxidants reduced substrate damage, although significant degradation of *N*-Ac-Ala-OH was observed in all cases. This suggests that a noticeable amount of substrate is degraded by direct reaction with hydroxyl radicals rather than by the chain reaction of autoxidation. Antioxidants would be expected to break autoxidation chains more efficiently than to intercept hydroxyl radicals. Tea extracts (EC and EGCG) appear to be more efficient antioxidants under reaction conditions than rosemary extracts (RA and CA). EGCG provides the best protection against oxidation. CA is a relatively poor preventive antioxidant as it has only one position in the aromatic ring available for hydroxylation; this explains its weaker performance in our system and is consistent with the other observations that it acts predominantly as a chain-breaking rather than a preventive antioxidant.

EC and ECGC were the best performing antioxidants in our system. This can be attributed to the available positions in the aromatic rings for hydroxylation but also their excellent ability to act as chain-breaking antioxidants as they have very weak benzylic C-H and phenolic O-H bonds. Finally, rosmarinic acid performed less well, potentially due to the lack of labile C-H bonds. We note that no hydroperoxides were observed for rosmarinic acid, consistent with the lack of C-H hydrogen abstraction.

## 3. Materials and Methods

### 3.1. Chemicals

Unless otherwise stated, all reagents and solvents were purchased from commercial sources and used without further purification. *N*-Ac-Ala-OH (≥97%) was obtained from Fluorochem, Hadfield, UK. Three catechol-based antioxidants (EC, RA and CA) were supplied as isolated compounds (powders) by 1Pluschem, San Diego, US; EGCG was purchased from Sigma-Aldrich, St. Louis, US. For all antioxidants, fresh stock solutions in a 1:1 ratio of MeOH:H_2_O were prepared on the day of irradiation. All experiments and aqueous solutions were prepared using Milli-Q water, or deuterium oxide (D_2_O) that was supplied by Sigma-Aldrich, Dorset, UK. Hydrogen peroxide (30 wt.%,) was obtained from Fischer Chemical, Loughborough, UK. Water (LC-MS grade, ≥99.9%, Fischer Chemical, Loughborough, UK), acetonitrile (LC-MS grade, ≥99.9%, Fischer Chemical, Loughborough, UK), formic acid (LC-MS grade, ≥99%, Fischer Chemical, Loughborough, UK) and formic acid-d_2_ (95 wt.% in D_2_O, 98% atom % D, Sigma-Aldrich, Dorset, UK) were used for MS characterisation. Glassware was cleaned in concentrated nitric acid and thoroughly rinsed with Milli-Q water. This procedure was found to be essential for the removal of traces of iron [48].

### 3.2. HO^•^-Mediated Oxidation of N-Ac-Ala-OH in the Presence of Antioxidants

Aqueous solutions of *N*-Ac-Ala-OH (1 mM) were exposed to UV light in the presence of aqueous H_2_O_2_ (100 mM), and antioxidant (0.1 mM), using a Philips HPK 125 W high pressure Hg lamp (Philips, Farnborough, UK) with a H_2_O filter (5 cm) which provides broad-band UV light [49,50]. The light output from this high pressure Hg lamp provides maximum energy at 365 nm with substantial radiation also at 435, 404, 313 and 253 nm [51]. All irradiations were carried out in H_2_O or D_2_O. Irradiations were carried out in a quartz cuvette (3 mL final reaction volume; open to air). The glassware was placed 10 cm in front of the UV lamp. Samples were exposed to UV light for 2 min and were analysed (undiluted) by LC-MS (4.3) immediately after cessation of irradiation.

### 3.3. LC-MS Analysis

Separation of irradiated samples was performed using LC-MS using an Agilent 1200 liquid chromatography machine (Agilent, Stockport, UK) equipped with a reverse phase 2.7 μm Waters Correct T3 (150 × 3 mm) column (Waters, Wilmslow, UK) and coupled to a SolariX XR FTMS 9.4T mass spectrometer (Bruker, Durham, UK). The mass spectrometer was calibrated daily using a dilute solution of sodium trifluoroacetate (NaTFA) in a 1:1 MeCN:H_2_O mixture in ESI positive ion mode. Samples were separated at 25 °C with a flow rate of 0.2 mL min^−1^ by gradient elution (Table 1), and 1 scan with a 0.2 s accumulation time. Elution solvents were: (A) 0.1% formic acid in H_2_O and (B) 0.1% formic acid in MeCN. When irradiations were carried out in D_2_O (instead of H_2_O), elution solvents were: (A) 0.1% formic acid-d_2_ in D_2_O, and (B) 0.1% formic acid-d_2_ in MeCN. ESI settings were as follows: drying gas flow = 4.0 L min^−1^; nebulizer pressure: 2.0 bar; dry temperature: 240 °C, capillary voltage = 4500 V; spray shield voltage = −500 V; skimmer voltage = 15 V. 

### 3.4. N-Ac-Ala-OH LC-MS Calibration Curve

To assess the HO^•^-derived decay of *N*-Ac-Ala-OH in the presence and absence of the antioxidants, an LC-MS calibration curve was constructed by separating the parent peptide using LC-MS (4.3) with a gradient elution (4.3; Table 1). The calibration curve was constructed by preparing the most concentrated sample of the substrate (1 mM), and then preparing serial dilutions (0.2–0.8 mM). The method afforded an excellent calibration curve (R^2^ > 0.997). The total ion count (TIC) of the [M + H]^+^ extracted ion chromatogram (EIC) was used. Daily and weekly variations of the calibration curves were assessed by repeating LC-MS injections with fresh solutions for all diluted samples.

## 4. Conclusions

Radical capture by antioxidants may not be relevant for living systems where the redox balance is determined by other processes. However, in dead biological tissue such as hair or wool, the ability of antioxidants to reduce oxidative damage is determined by their chemical reactions with reactive radicals. In order to improve our understanding of antioxidant action in these systems, we investigated the preventive and chain-breaking properties of polyphenolic antioxidants found in tea and rosemary. Epicatechin, epigallocatechin gallate, rosmarinic and carnosic acids (pure compounds) were studied in the oxidation of *N*-Ac-Ala-OH with photochemically generated hydroxyl radicals. The formation of hydroxylated products, quinones and hydroperoxides can be correlated with the antioxidant activity. Carnosic acid only has one position for hydroxylation. It was the weakest antioxidant in this study, presumably due to its relatively poor performance as a preventive antioxidant. Rosmarinic acid did not form any hydroperoxides, presumably because it lacks labile C-H benzylic bonds. The two catechins showed the best performance; they can react with both hydroxyl and peroxyl radicals as quinones, and hydroxylated derivatives and hydroperoxides were detected for all these compounds.

## Figures and Tables

**Table 1:** Gradient elution for LC-MS separation. Elution solvents consisted of (A) 0.1% formic acid in H_2_O and (B) 0.1% formic acid in MeCN.

Time/min	% A	% B
0	100	0
5	100	0
10	70	30
15	70	30
25	50	50
35	50	50
40	30	70
45	30	70
50	100	0
60	100	0

## Data Availability

All data are available within the manuscript.

## Supplementary Materials

The following supporting information can be downloaded at: https://www.mdpi.com/article/10.3390/molecules28227514/s1, Figure S1. UV-Vis Spectra of the N-Ac-Ala-OH (1 mM) and the four antioxidants (all 0.1 mM) used for this study; Figure S2. UV-Vis Spectra of the reaction mixtures of N-Ac-Ala-OH (1 mM) and EGCG (0.1 mM), and N-Ac-Ala-OH (1 mM) and RA (0.1 mM), before and after exposure to UV light; Figure S3. Base peak (BPC) and extracted ion (EIC, m/z 291.08312) HPLC-MS chromatograms in +ve ESI mode for EC (0.1 mM, top), and the +ve mode ESI MS of EC (bottom); Figure S4. Base peak (BPC) and extracted ion (EIC, m/z 457.07708) HPLC-MS chromatograms in -ve ESI mode for EGCG (0.1 mM, top), and the -ve mode ESI MS of EGCG (bottom); Figure S5. Base peak (BPC) and extracted ion (EIC, m/z 331.19150) HPLC-MS chromatograms in -ve ESI mode for CA (0.1 mM, top), and the -ve mode ESI MS of CA (bottom); Figure S6. Base peak (BPC) and extracted ion (EIC, m/z 359.07746) HPLC-MS chromatograms in -ve ESI mode for RA (0.1 mM, top), and the -ve mode ESI MS of RA (bottom); Figure S7. Potential hydroxylated derivatives generated during the hydroxyl radical-mediated oxidation of the four antioxidants during the irradiations.
