# Supplementary material for: The Role of Polyphenolic Antioxidants from Tea and Rosemary in the Hydroxyl Radical Oxidation of N-Acetyl Alanine
Source: Molecules. 2023 Nov 10;28(22):7514. doi: 10.3390/molecules28227514 (PMC10673243; doi:10.3390/molecules28227514)
Supplement: Supplementary file 1 [file molecules-28-07514-s001.zip › molecules-2679491-supplementary.pdf]

Supplementary Materials: The Role of Polyphenolic Antioxidants from Tea and Rosemary in the Hydroxyl Radical Oxidation of *N*-Acetyl Alanine

Nikolaos Vagkidis, Jennifer Marsh, Victor Chechik

Contents:

1. UV-Vis Spectra of pure compounds and reaction mixtures before and after UV irradiation.

Figure S1. UV-Vis Spectra of the *N*-Ac-Ala-OH (1 mM) and the four antioxidants (all 0.1 mM) used for this study. 2

Figure S2. UV-Vis Spectra of the reaction mixtures of N-Ac-Ala-OH (1 mM) and EGCG (0.1 mM), and N-Ac-Ala-OH (1 mM) and RA (0.1 mM), before and after exposure to UV light. 3

2. HPLC-MS chromatograms of the four antioxidants

Figure S3. Base peak (BPC) and extracted ion (EIC, m/z 291.08312) HPLC-MS chromatograms in +ve ESI mode for EC (0.1 mM, top), and the +ve mode ESI MS of EC (bottom). 4

Figure S4. Base peak (BPC) and extracted ion (EIC, m/z 457.07708) HPLC-MS chromatograms in -ve ESI mode for EGCG (0.1 mM, top), and the -ve mode ESI MS of EGCG (bottom). 5

Figure S5. Base peak (BPC) and extracted ion (EIC, m/z 331.19150) HPLC-MS chromatograms in -ve ESI mode for CA (0.1 mM, top), and the -ve mode ESI MS of CA (bottom). 6

Figure S6. Base peak (BPC) and extracted ion (EIC, m/z 359.07746) HPLC-MS chromatograms in -ve ESI mode for RA (0.1 mM, top), and the -ve mode ESI MS of RA (bottom). 7

3. Possible chemical structures for the hydroxylated products of the four antioxidants.

Figure S7. Potential hydroxylated derivatives generated during the hydroxyl radical-mediated oxidation of the four antioxidants during the irradiations. 8

1. UV-Vis Spectra of pure compounds and reaction mixtures before and after UV irradiation.

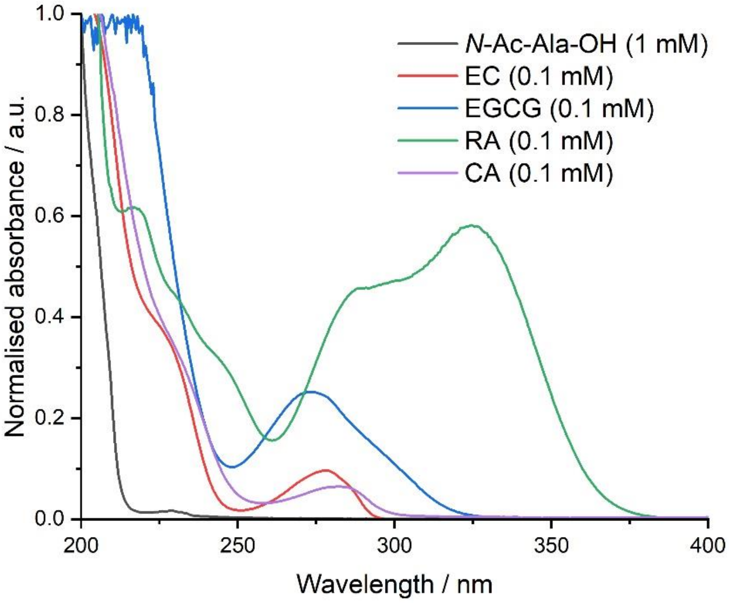

Figure S1. UV-Vis Spectra of the *N*-Ac-Ala-OH (1 mM) and the four antioxidants (all 0.1 mM) used for this study.

EGCG and RA showed a considerable absorbtion at high wavelenghts (i.e., > 270 nm). Therefore, to assess whether their direct photoexcitation from the UV lamp could influence their photochemistry, these two compounds were further examined in the absence of H_2O_2. Their aqueous solutions (0.1 mM) were exposed to UV light in the presence of *N*-Ac-Ala-OH (1 mM) for 10 min. Figure S2 shows the UV spectra of the reaction mixtures before any exposure to UV light, and after 10 min of irradiation.

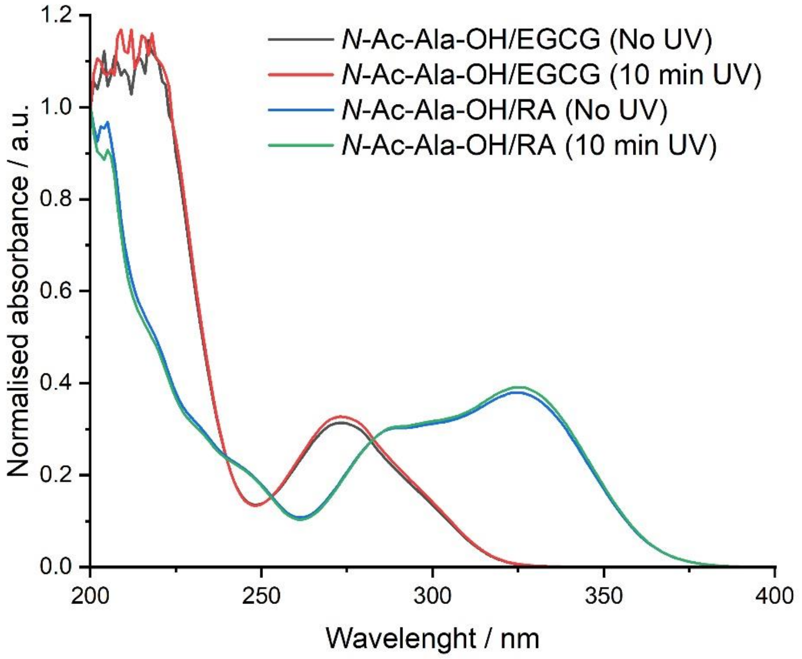

Figure S2. UV-Vis Spectra of the reaction mixtures of *N*-Ac-Ala-OH (1 mM) and EGCG (0.1 mM), and *N*-Ac-Ala-OH (1 mM) and RA (0.1 mM), before and after exposure to UV light.

2. HPLC-MS chromatograms of the four antioxidants

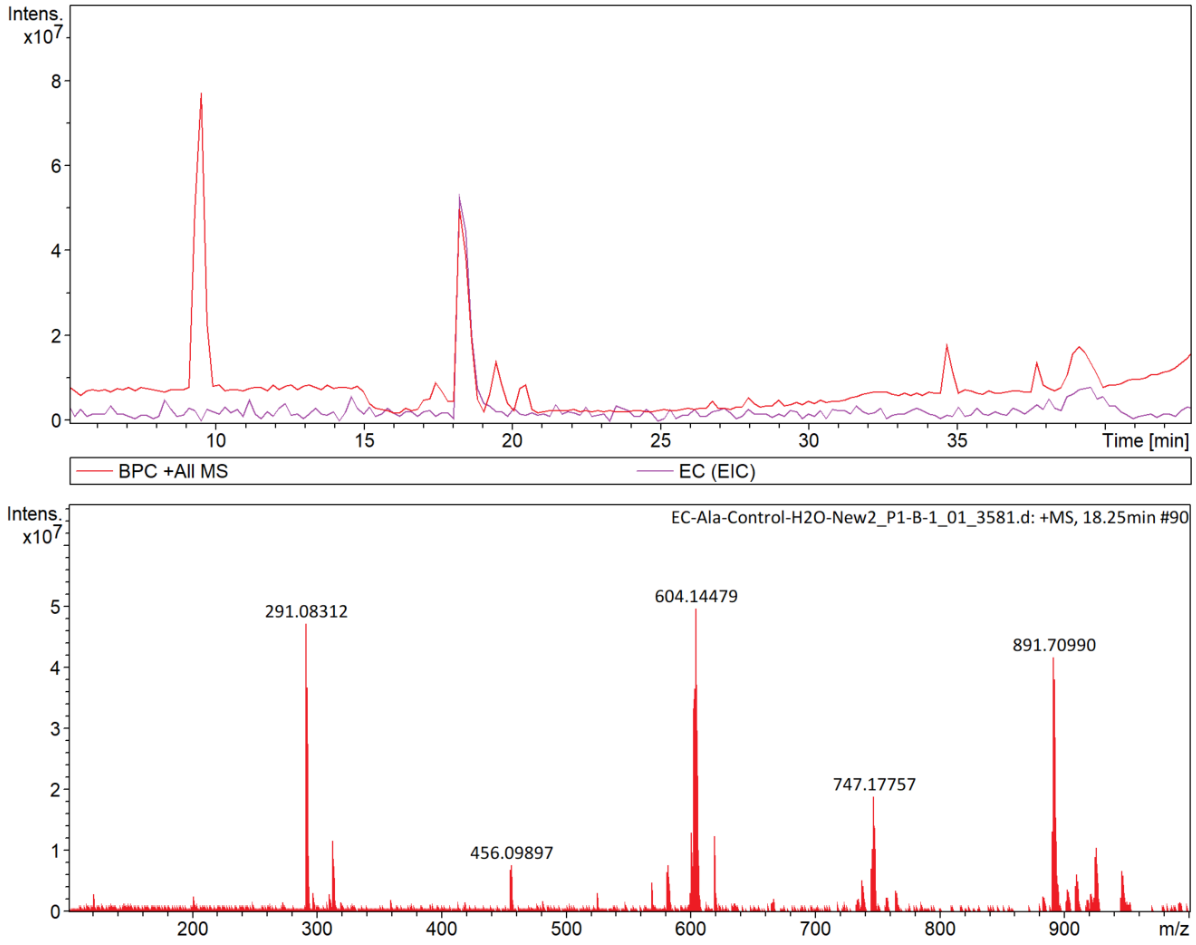

Figure S3. Base peak (BPC) and extracted ion (EIC, m/z 291.08312) HPLC-MS chromatograms in +ve ESI mode for EC (0.1 mM, top), and the +ve mode ESI MS of EC (bottom).

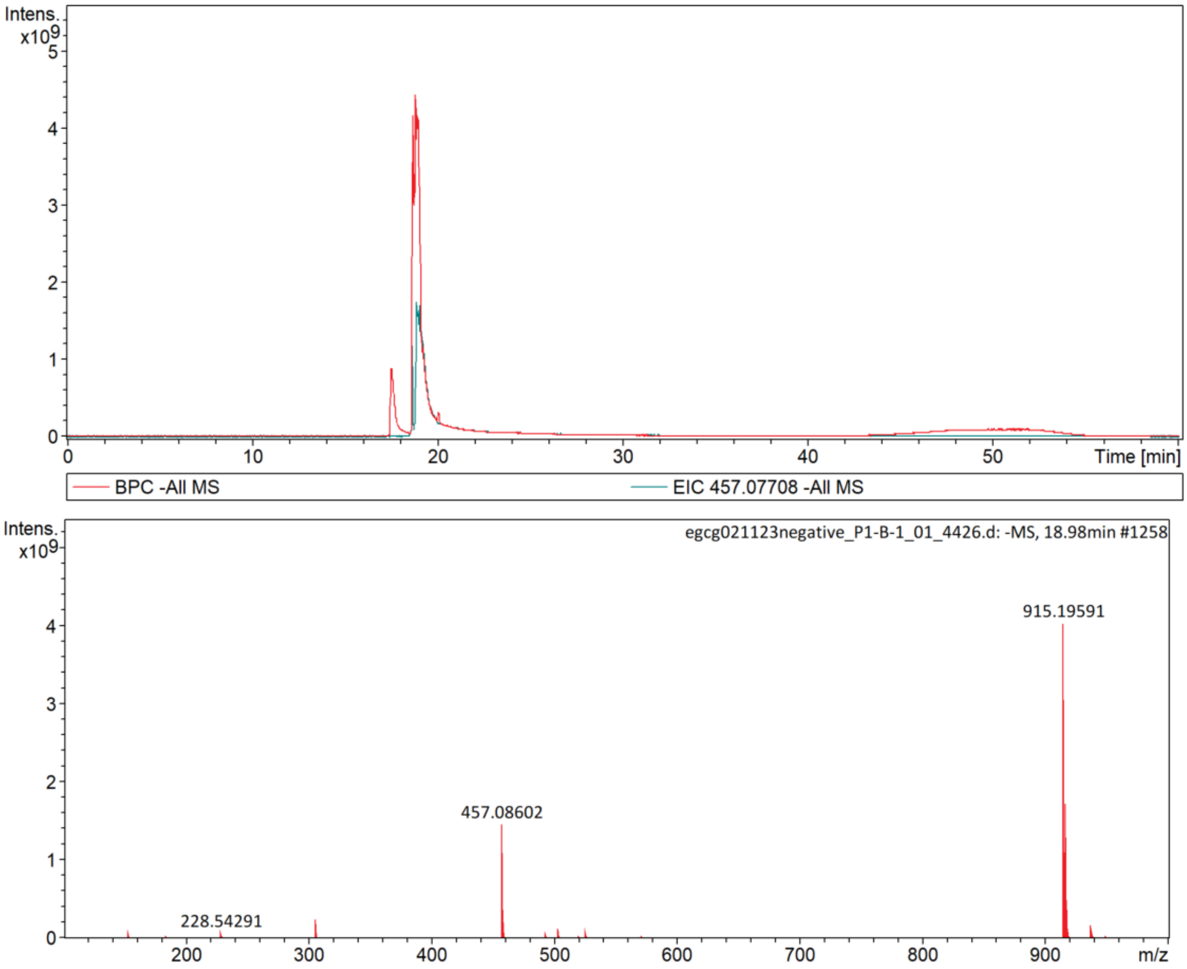

Figure S4. Base peak (BPC) and extracted ion (EIC, m/z 457.07708) HPLC-MS chromatograms in -ve ESI mode for EGCG (0.1 mM, top), and the -ve mode ESI MS of EGCG (bottom).

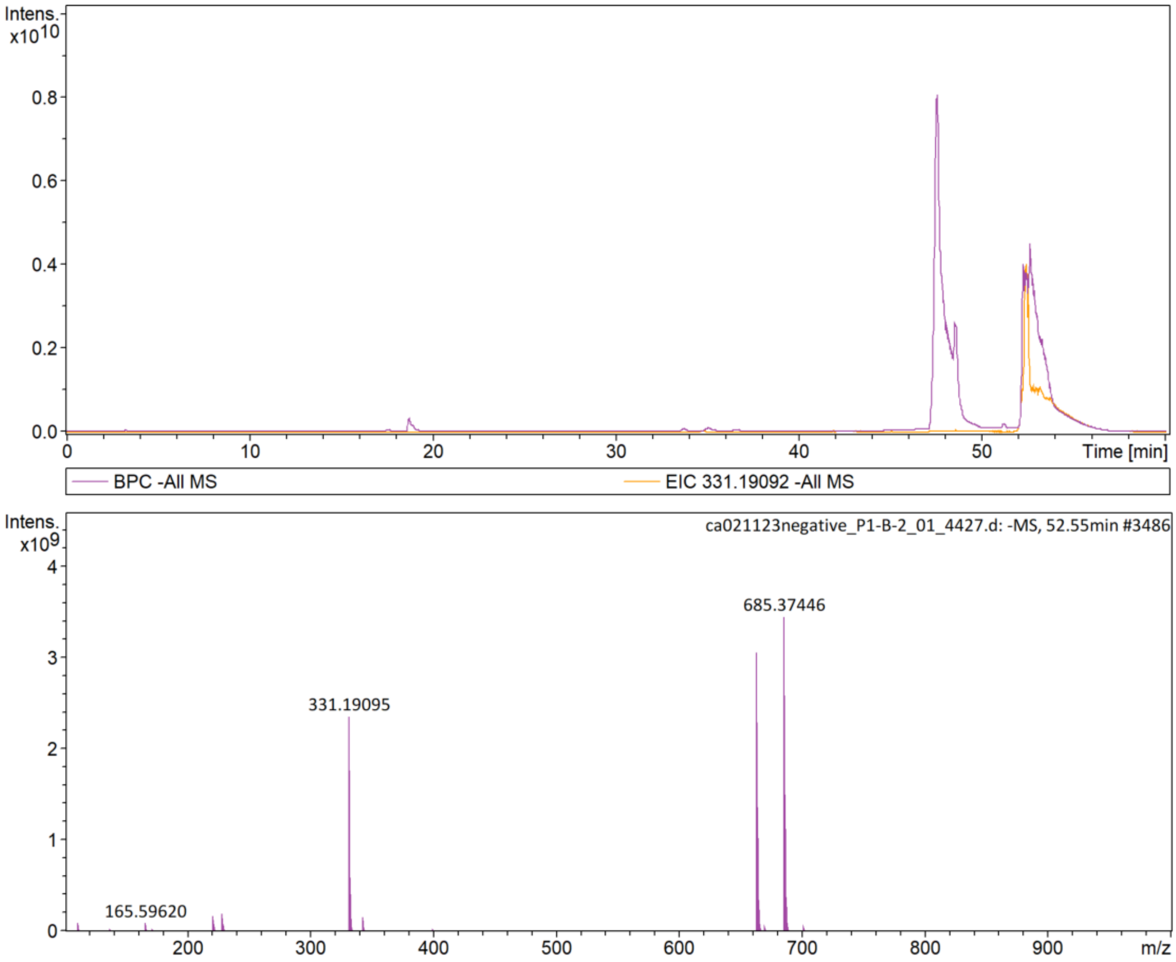

Figure S5. Base peak (BPC) and extracted ion (EIC, m/z 331.19150) HPLC-MS chromatograms in -ve ESI mode for CA (0.1 mM, top), and the -ve mode ESI MS of CA (bottom). The peak eluting at ca. 48 min in HPLC is carnosol.

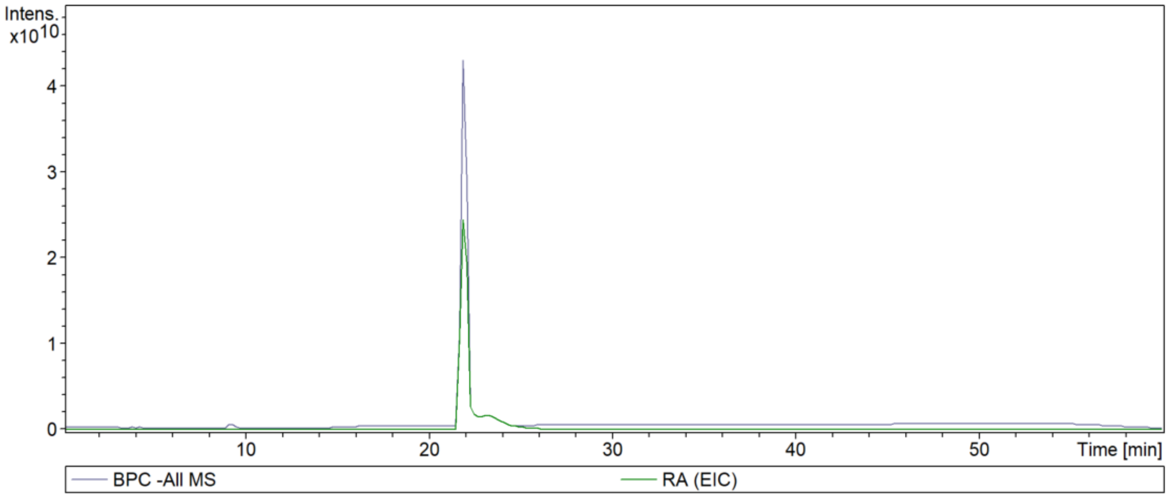

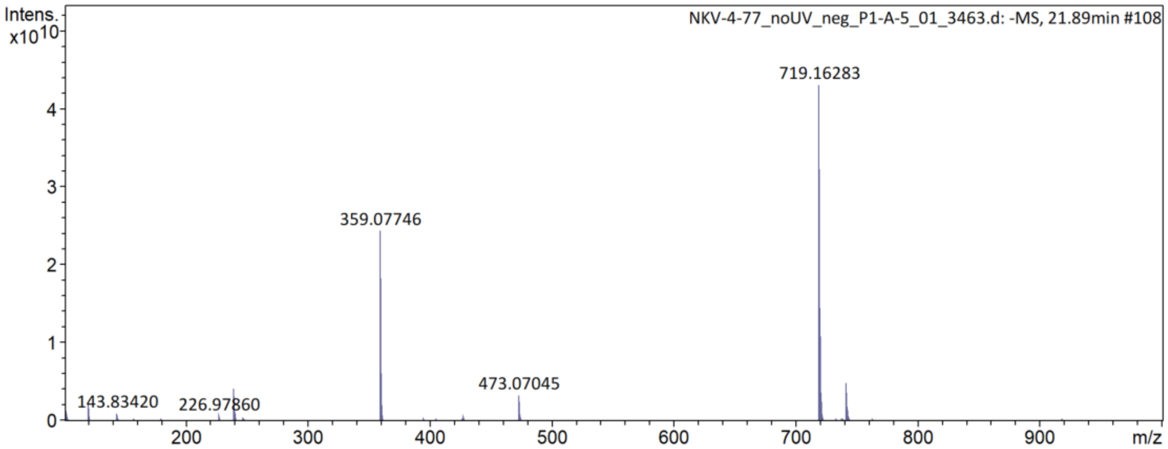

Figure S6. Base peak (BPC) and extracted ion (EIC, m/z 359.07746) HPLC-MS chromatograms in -ve ESI mode for RA (0.1 mM, top), and the -ve mode ESI MS of RA (bottom).

3. Possible chemical structures for the hydroxylated products of the four antioxidants.

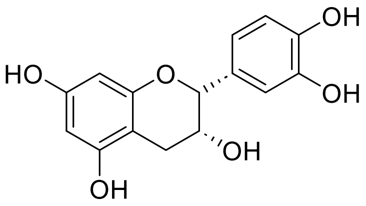

Epicatechin (EC)

4 possible and 1 less likely hydroxylated isomers

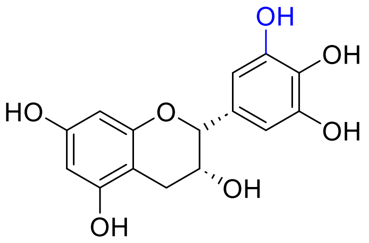

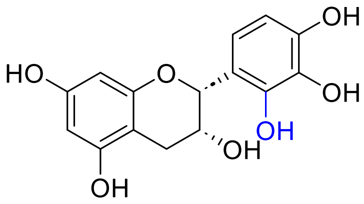

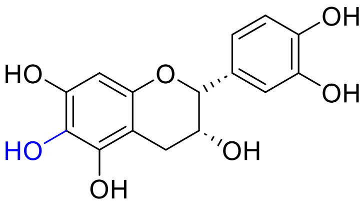

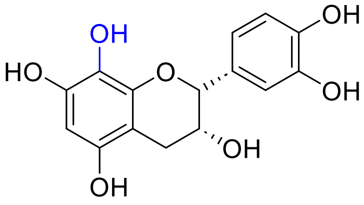

Not activated by adjacent OH; less likely:

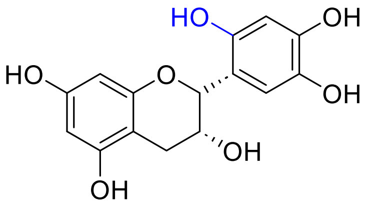

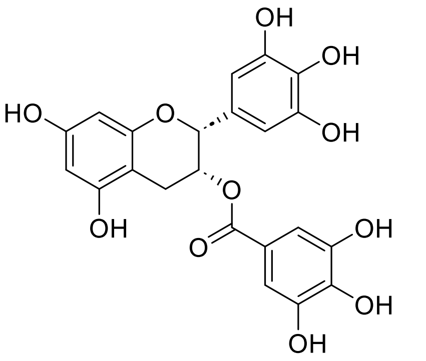

Epicagallocatechin gallate (EGCG)

6 possible hydroxylation isomers

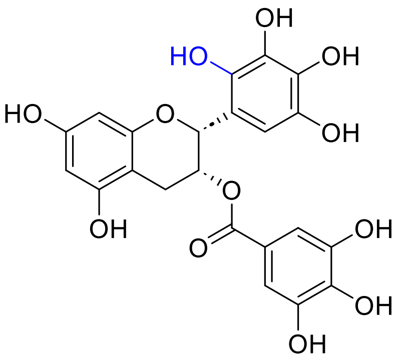

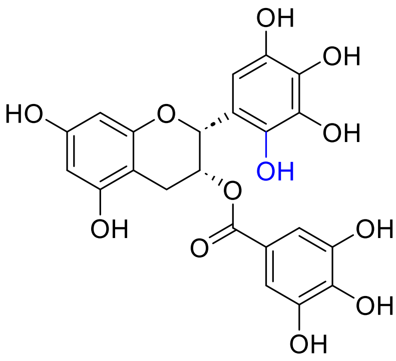

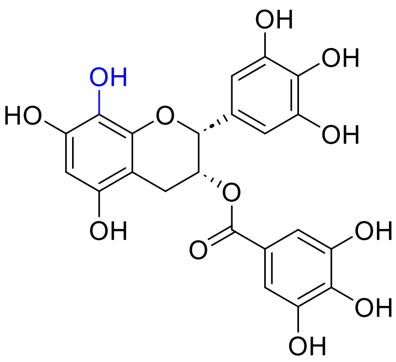

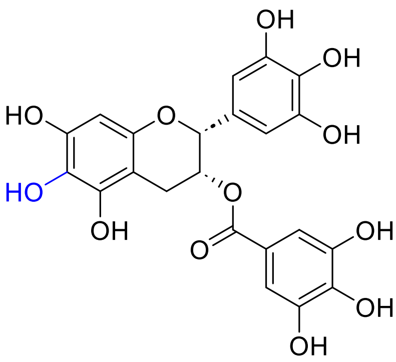

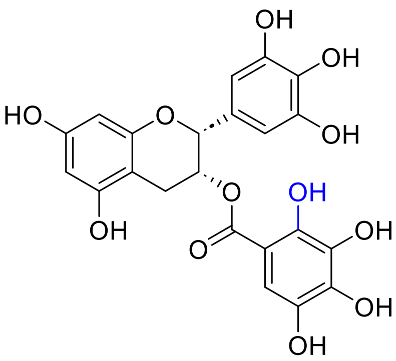

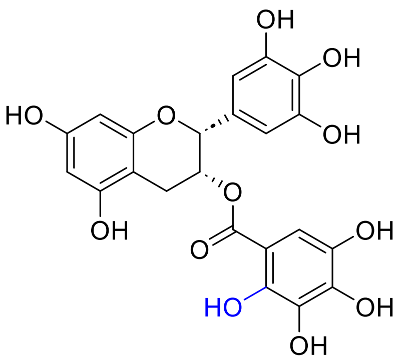

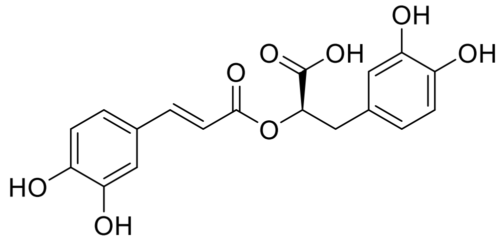

Rosmarinic acid (RA)

4 possible and 2 less likely hydroxylation isomers

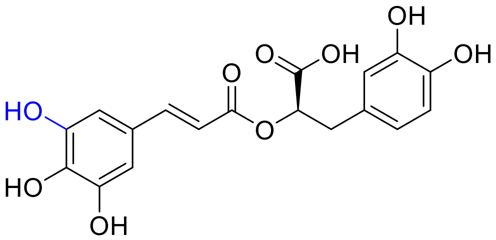

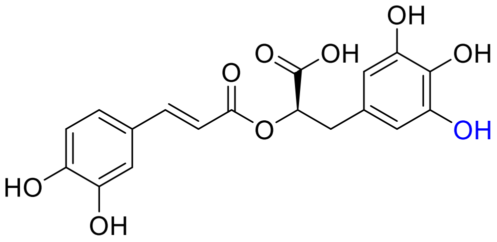

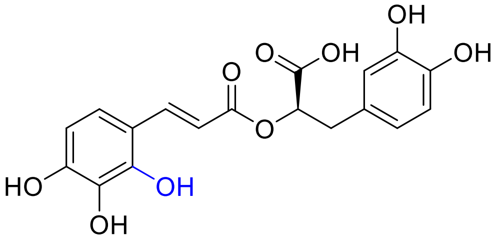

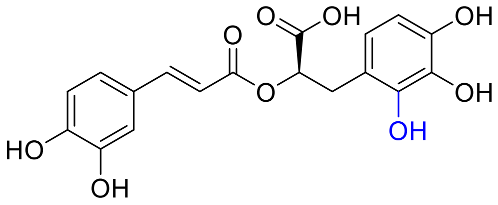

Not activated by adjacent OH, less likely:

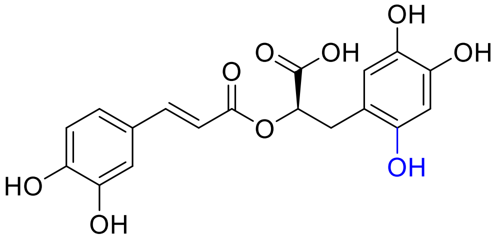

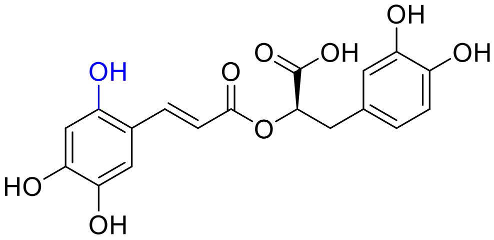

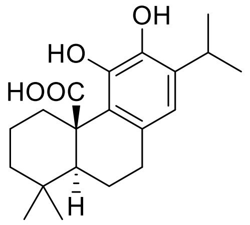

Carnosic acid (CA)

1 possible isomer

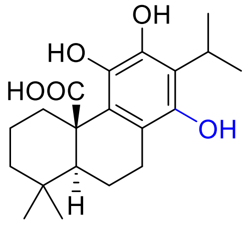

Figure S7. Potential hydroxylated derivatives generated during the hydroxyl radical-mediated oxidation of the four antioxidants during the irradiations.
